# Enriched‐biochar application increases broccoli nutritional and phytochemical content without detrimental effect on yield

**DOI:** 10.1002/jsfa.12102

**Published:** 2022-07-28

**Authors:** Daniel Montoya, Juan Antonio Fernández, José Antonio Franco, María del Carmen Martínez Ballesta

**Affiliations:** ^1^ Ingeniería Agronómica Universidad Politécnica de Cartagena Cartagena Spain; ^2^ Recursos fitogenéticos Instituto de Biotecnología Vegetal, Edificio I + D + i Cartagena Spain

**Keywords:** biochar, broccoli, glucosinolates, minerals, phenolic compounds

## Abstract

**BACKGROUND:**

Soil fertility is a major concern during vegetable production. Conventional *versus* organic fertilization has been studied in order to conserve soil properties. While some reports point out an increase in food nutritional properties, the loss of crop yield under organic conditions continues to be a problem. Thus, an experiment with broccoli in the field was carried out, comparing crop management under conventional fertilization (CF) and two soil amendment treatments: manure pellet (M) and an enriched‐biochar (EB) supplemented by an organic fertilizer (AND) applied alone (M + CF; EB+AND) or in combination (M + EB + AND). Crop yield and the nutritional properties in the flowering heads (mineral content, phenolic compounds and glucosinolates (GSLs)), were determined.

**RESULTS:**

Enriched‐biochar and manure as a standalone amendment resulted in higher crop yield regarding CF, but not when they were applied in combination. The number of flowering heads with no‐commercial characteristics was lower after enriched‐biochar soil application. Finally, enriched‐biochar treatment enhanced NO_3_^−^, PO_4_^3−^ and SO_4_^2−^ levels in the flowering heads, and some of the ion contents can be associated with mineral changes in the soil after the biochar amendment. Also, the contents of phenolic compounds and indole GSLs were higher after enriched‐biochar application compared with the other treatments, GSL increase being due to the higher percentage of sulfur in the plant rather that an adequate N/S ratio.

**CONCLUSION:**

Application of enriched‐biochar amendment in the cultivation of broccoli is appropriate, since there are no losses of yield and an increase in nutritional compounds in the flowering heads. © 2022 The Authors. *Journal of The Science of Food and Agriculture* published by John Wiley & Sons Ltd on behalf of Society of Chemical Industry.

## INTRODUCTION

Long‐term intensive agriculture reduces soil fertility and therefore the ability to support the growing population.^1^ Climatic change may aggravate soil deterioration, and the development of sustainable agricultural practices requires special attention.

Different strategies have been applied to recover undesirable effects on soil fertility as well as to mitigate climatic change. ‘Terra preta’ soils, with a porous physical structure, are a potential tool for both mitigating climate change and sustainably increasing agricultural productivity.^2^ Soil organic matter of terra preta is composed of up to 35% black carbon, which is the main component of biochar,^3^ a charcoal proceeding from vegetable biomass wastes. Thus the use of biochar has become of interest^4^ since it may improve soil physical properties by favouring soil aggregation,^5^ aeration^6^ and water retention.^7^

It has been observed that biochar amendment has the potential for scavenging carbon into its pores. In addition, it reduced soil N_2_O emissions, balanced the pH levels in acidic soils and increased nutrient retention.^4^, ^7^, ^8^, ^9^ Biochar has proven positive effects on nutrient retention, increasing cation‐exchange capacity (CEC) and water‐holding capacity,^10^ and soil microbial and mycorrhizal activity.^11^, ^12^ Thus the addition of biochar (10%) with urea raised the nitrogen use efficiency (NUE) in wheat plants.^13^ Ahmed *et al*. observed that biochar loaded with P improved the bioavailability of P in the soil and plants.^14^ Finally, the increase of Si uptake by the plants was also stimulated by the addition of biochar mixed with KOH (10%).^15^

Regarding plant productivity and growth, an increase in the biomass production in *Zea mays* plants treated with coconut husk biochar has been observed.^16^ Also, bamboo biochar induced growth and nodulation in soybean.^17^ Furthermore, biochar has been applied to stress management, ameliorating adverse stress effects on plant growth. For example, sunflower plants increased their biomass under water‐deficient conditions after biochar treatment, through enhanced water use efficiency.^18^ In contaminated soils, biochar resulted in an efficient amendment to improve plant physiology under metal stress conditions.^19^ However, Vaccari *et al*. indicated that the effect of biochar on agricultural productivity depended on plant species or the targeted part of the plant.^20^ They showed that application of biochar at 14 t ha^−1^ increased vegetative growth of tomato plants, but not fruit yield.^20^ Furthermore, biochar application could also result in delay of flowering for legume plants.^21^

Despite the high number of reports concerning the benefits of biochar on soil microbiome or plant growth and development, there is a lack of information about the effects of biochar on the bioactive compounds in the plant. In one report, olive tree pruning‐derived biochar combined with mineral fertilizers enhanced glucosinolate (GSL) levels in broccoli plants.^22^ Khalid *et al*. observed that combined biochar with organic fertilization increased the level of flavonoids in Chinese cabbage.^23^ Also, lettuce crops grown in soil moderately contaminated with copper, increased total phenolic compounds and anthocyanins after the addition of biochar derived from orchard pruning feedstock.^24^

However, there are also some contradictory studies where biochar had negative impacts on plant growth and soil aging, while the addition of fresh biomass might be required for optimal soil fertility and microbiome recovery.^25^

The aim of this study was to test the effect of enriched‐biochar application on plant yield and nutritional composition in broccoli plants. For that purpose the biochar supply was realized in a semiarid area of southeast Spain, and its ability to improve the bioactive compounds in the flowering heads was compared with other organic amendments such as manure. Plant yield, mineral content, phenolic compounds and GSL were analysed in broccoli heads after biochar application, comparing these parameters with conventional fertilization and the application of manure alone or in combination with enriched‐biochar to study synergistic effects.

## MATERIAL AND METHODS

### Field experiment

Seeds of broccoli (*Brassica oleracea* L. Italica group) cultivar Bacano were sown in the shading screen nursery in foam trays (84 cells), using a mixture of peatmoss and vermiculite (1:1, v/v) as substrate. Seedlings were planted in a field trial (CDTA‐El Mirador station, Murcia, Spain) (37° 50′ 51.9″ N, 0° 53′ 00.1″ W) after 39 days from seed sowing, when plants were a three‐ to four‐leaf stage.

**Figure 1:**
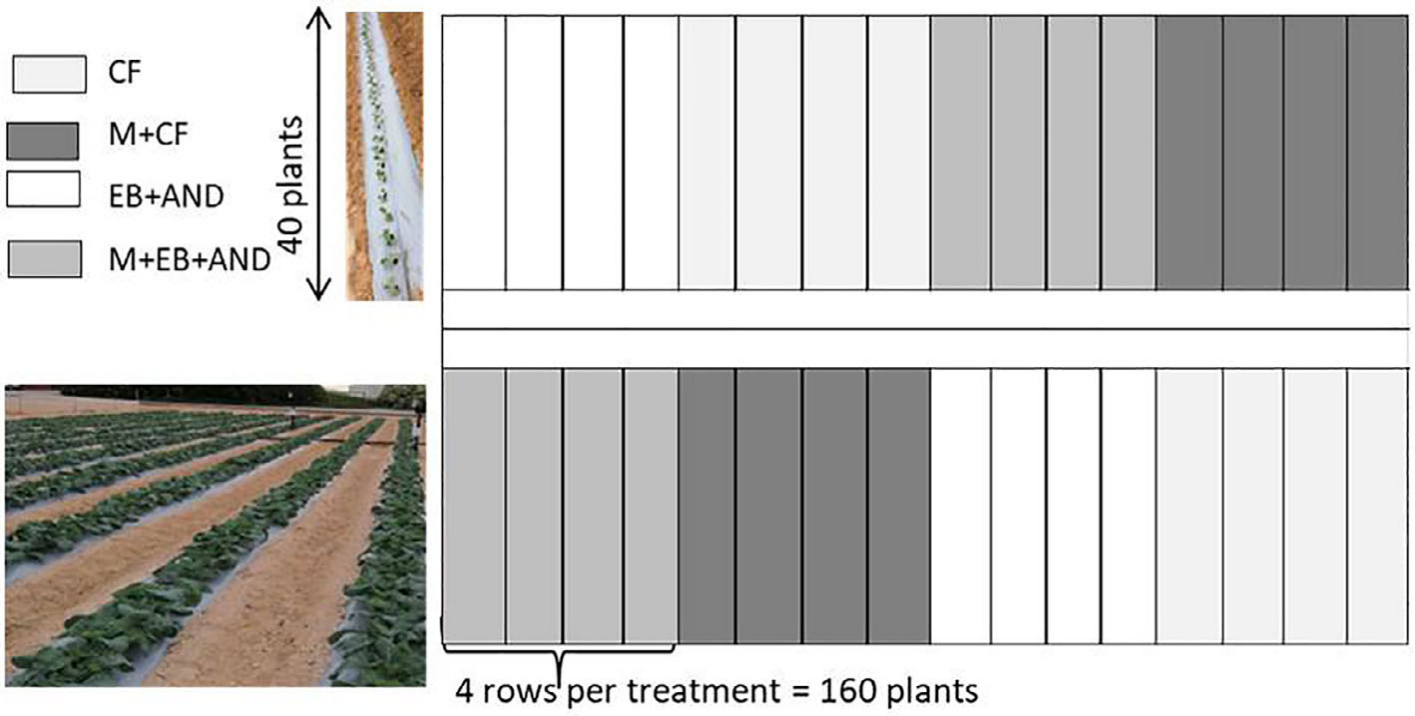
Experimental design at CDTA‐El Mirador station, Murcia, Spain.

The experimental test lot was divided into random blocks of four rows with a total of two repetitions per treatment (Fig. 1). This makes a total of eight test subplots with 40 plants to each subplot. The surface of each subplot was 64 m^2^ – a total of 128 m^2^ per treatment and 160 plants per repetition. Broccoli plants were transplanted on 14 October 2019 with a spacing of 100 cm between rows and 20 cm between plants. The crop was harvested twice according to the commercial size of the flowering heads (16 and 24 January).

**Figure 2:**
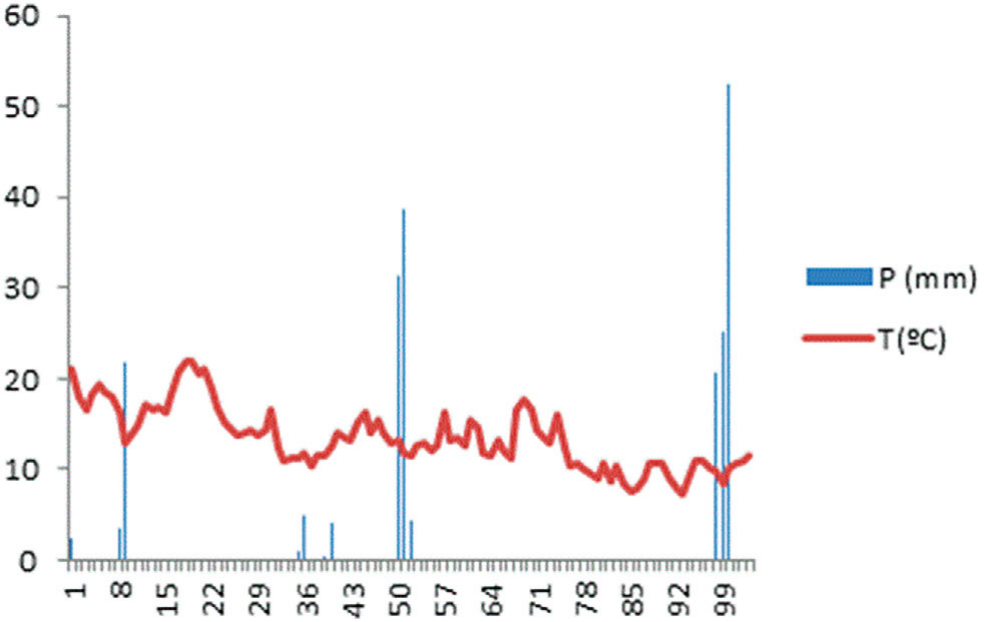
Average daily temperature (red) and precipitation (blue) during the experimental season (October–December, 2019).

During the experimental period, rainfall and air temperature were recorded daily at a meteorological station at the experimental site. Average air daily temperature and precipitation during the growth season are shown in Fig. 2 December and November presented were the months with higher precipitation (mean 98.8 and 75.2 mm, respectively), while mean temperature remained similar during the growing season.

The treatments were the following: (CF): conventional fertilization (Supporting Information, Table S1). (CF+M): conventional fertilization and manure pellet as amendment (Supporting Information, Table S2). (EB+AND): enriched‐biochar (EB) (Supporting Information, Table S3) and organic fertilization (AND) (Supporting Information, Table S4) as amendment, (M + EB + AND): manure (M) and enriched‐biochar (EB) as amendments with organic fertilization (AND). Manure and enriched‐biochar were applied before transplanting.

To obtain the samples, broccoli pieces were collected from the two central lines of each repetition and they were evaluated separately.

### Yield and production

The flowering heads were collected when they were of commercial size with the buds of the head firm and tight, and with a fixed head length (average 200 mm), stalk diameter (average 150–180 mm) and stem diameter (30–50 mm). The total fresh weight (FW) was directly recorded with portable scales (PCE‐EP 1500, PCE‐Iberica, Tobarra, Spain) in the cooperative farms to calculate stalk fresh weight (FW). Heads classification was the following according to the framer's criteria (Supporting Information, Table S5). The plant yield was determined as kg m^−2^ for individual and total recollection dates.

### Leaf fresh and dry weight

Leaves biomass was determined before harvest after 12 weeks of transplanting. Plant parts (leaves and flowering heads) were harvested and weighed to determine fresh canopy mass (FWc). They were then oven dried at 70 °C for 48 h and dry weight (DWc) was measured using a digital balance (model RADWAG PS 4500/C2, Radwag España balanzas, Lorca, Murcia, Spain) with an accuracy of 0.001 g.

### Anion and cation analysis

Anions and cations were extracted from the ten samples per treatment and replicates. For that, 0.2 g of leaf and stalk dry tissues were used and 50 mL distilled water was added. Then, the tubes were shaken in an orbital shaker (Stuart SSL1, Stone, St Neots,Cambridgeshire, UK) for 45 min at 110 rpm at 50 °C. The ion content was quantified by ion chromatography using a Metrosep A SUPP 5 column (Metrohm AG, Zofingen, Switzerland) with a flow rate of 0.7 mL min^– 1^ for anions and a Metrosep C 2–250 column (Metrohm AG) with a flow rate of 1.0 mL min^– 1^ flow for cations.^26^

### Carbon and nitrogen analysis

The plants were dried in an oven at 80 °C for 72 h and samples were ground in a laboratory analytical mill (model IKA A10, IKA werke Gmbh & Co. KG, Staufen, Germany). The total carbon and nitrogen contents were analysed using a CN analyser (Thermo‐Finnigan 1112 EA elemental analyser; Thermo‐Finnigan, Milan, Italy).

### Extraction and determination of intact GSL and phenolic compounds

GSL and phenolic compounds were determined according to Dominguez‐Perles *et al*. (2010).^27^ For that, freeze‐dried powder (100 mg) of flowering head tissue was extracted in 1.5 mL of 70% methanol for 30 min at 70 °C, vortexed every 5 min to improve extraction and then centrifuged (20 min, 10 000 × *g*, 4 °C) (model Sigma 1–13, B Braun Biotech International, Osterode, Germany). Supernatants were collected and methanol was removed using a rotary evaporator. The dried residue was reconstituted in ultrapure water up to 1 mL and filtered through a 0.22 μm polypropylene membrane filter (ANOTOP 10 plus, Whatman, Maidstone, UK). Each sample (20 μL) was analysed in a Waters high‐performance liquid chromatography (HPLC) system (Waters Cromatografía SA, Barcelona, Spain) consisting of a W600E multisolvent delivery system, inline degasser, W717 plus autosampler and W2996 PAD. The compounds were separated in a Luna C18 column (25 cm × 0.46 cm, 5 μm particle size; Phenomenex, Macclesfield, UK) with a security guard C18‐ODS (4 × 30 mm) cartridge system (Phenomenex). The mobile phase was a mixture of water–trifluoroacetic acid (99.9:0.1, v/v) (A) and a mixture of acetonitrile–trifluoroacetic acid (99.9:0.1, v/v) (B). The flow rate was 1 mL min^−1^ in a linear gradient, starting with 1% B for 5 min to reach 17% B at 15 min, which was maintained for 2 min, then 25% B at 22 min, 35% B at 30 min, 50% B at 35 min and 99% B at 40 min. The monitored compounds, GSL (227 nm) and phenolic compounds (330 nm), were eluted from the column in 35 min. GSLs present in the samples were quantified using sinigrin as standard (sinigrin monohydrate from *Sinapis nigra*, Phytoplan Diehm & Neuberger GmbH, Heidelberg, Germany). Caffeoylquinic acid derivatives were quantified using chlorogenic acid (Sigma, St Louis, MO, USA), flavonoids with quercetin‐3‐rutinoside (Sigma) and sinapic acid derivatives using sinapinic acid (Sigma).^27^

### Statistics

The parameters adjusted to a normal distribution were subjected to one‐way analysis of variance (ANOVA). A multiple‐range Tukey's test was used to separate means, and statistical significance was assessed at the level *P* ≤ 0.05, using the SPSS 20.0 software package (IBM, Armonk, NY, USA).

## RESULTS

### Yield and production

**Figure 3:**
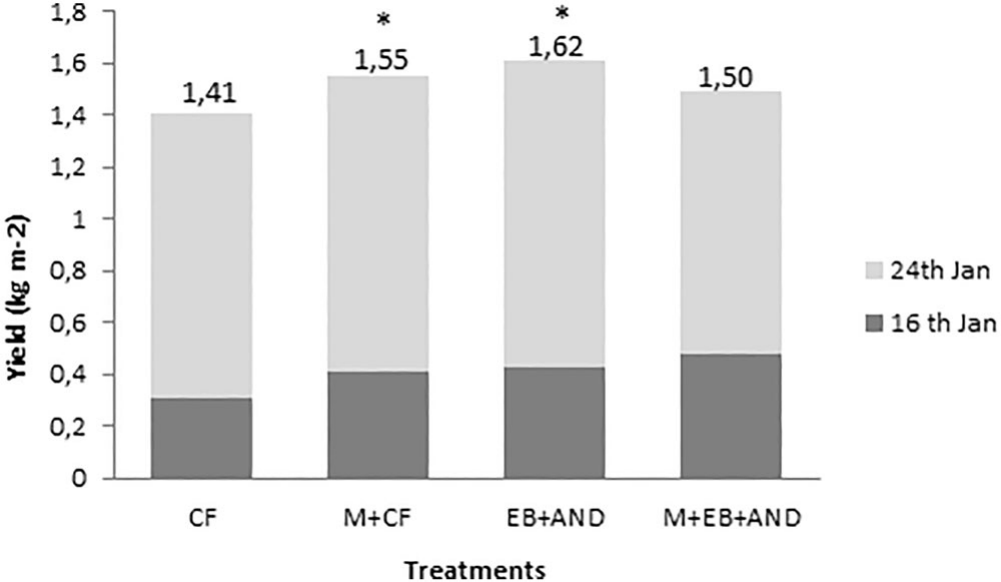
Total yield (kg m^−2^) at two recollection dates (16 and 24 January) of broccoli flowering heads harvested in the experimental parcels. CF, control plants with no organic amendment and conventional inorganic fertilization; M + CF: manure pellet amended soil and conventional fertilization; EB+AND: enriched‐biochar amended soil and organic fertilizer AND; M + EB + AND, manure pellet and enriched‐biochar amended soil treated with organic fertilization AND. Numbers show average value per treatment in the eight randomized subplots (*n* = 8) ± standard deviation. Different letters indicate statistically significant differences (*P* < 0.05).

The results indicated that total yield was significantly higher in M + CF and EB+AND treatments compared to CF (Fig. 3). No significant differences were found in the yield of M + CF‐treated plants regarding CF, in spite of the fact that in the first recollection this treatment resulted in a higher number and biomass of flowering heads. Thus the two amendment treatments (based on manure pellet and enriched‐biochar) significantly increased the total crop yield when they were applied individually, but it was not the case when both supplemented with AND fertilization (M + EB + AND) were combined.

**Figure 4:**
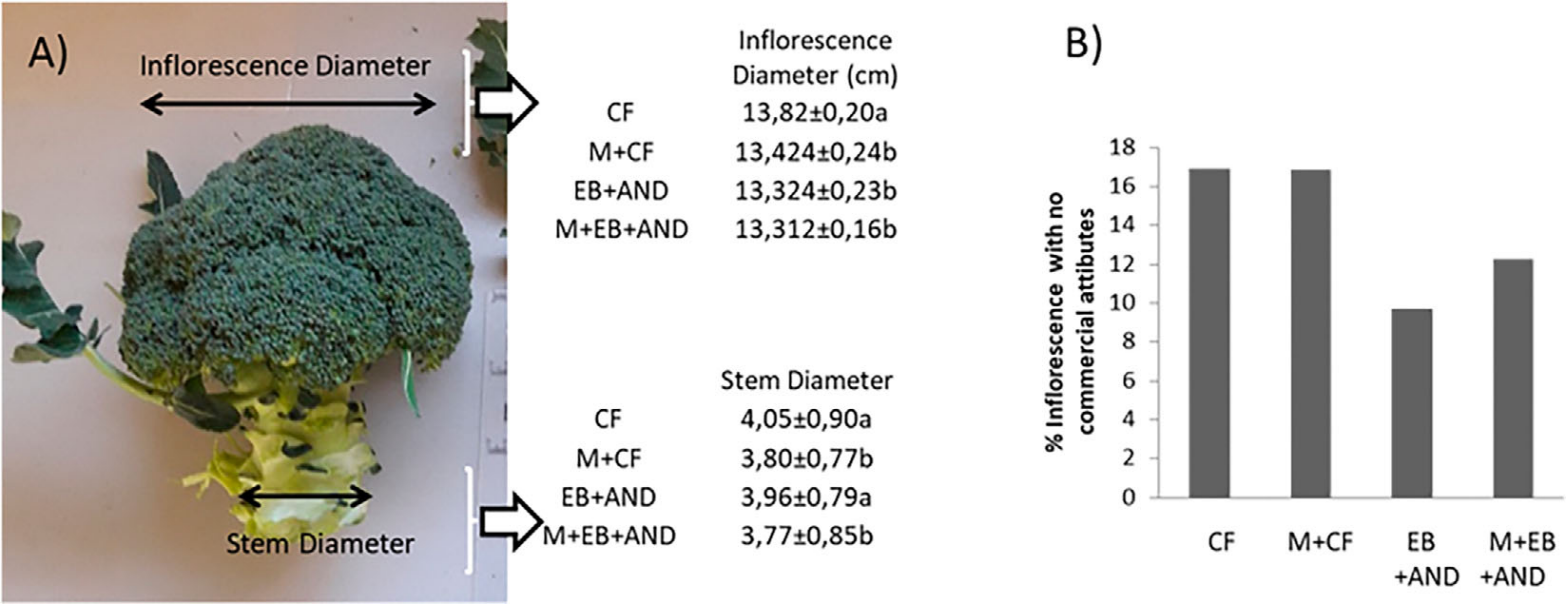
Percentage of flowering heads refused due to no‐commercial characteristics (A). External diameter and inner diameter of the harvested flowering heads at a fixed commercial length of 200 mm (B). CF, control plants with no organic amendment and conventional inorganic fertilization; M + CF, manure pellet amended soil and conventional fertilization; EB+AND, enriched‐biochar amended soil and organic fertilizer AND; M + EB + AND: manure pellet and enriched‐biochar amended soil treated with organic fertilization AND. Numbers show average value per treatment (*n* = 20) ± standard deviation. Different letters indicate statistically significant differences (*P* < 0.05).

Considering classification of the flowering heads by categories (Table 1), EB+AND and M + EB + AND treatments showed a higher number of first category flowering heads with regard to CF and M + CF, while these last treatments presented a higher number of second category flowering heads with regard to the two first treatments. Only significant differences were found between M + CF and M + EB + AND treatments in the fourth category flowering heads, the number being higher in the last one. Finally, EB+AND and M + EB + AND treatments resulted in a lower amount of industry category flowering heads in comparison to CF and M + CF. The diameters of the flowering heads and stems were determined (Fig. 4A). The flowering head diameter was significantly lower in all treatments in comparison with CF, but the diameter of the stems was similar in the EB‐AND and lower in the rest of the treatments compared to CF.

**Table 1:** Flowering head categories classification

Treatment	First	Second	Fourth	Industry	Flowering head FW (g)
CF	66.40 ± 2.51a	10.29 ± 1.59a	18.96 ± 3.0ab	7.39 ± 1.37a	514.80 ± 14.20a
M + CF	64.81 ± 6.54a	12.21 ± 2.96a	15.88 ± 3.53b	7.11 ± 2.37a	467.64 ± 17.55b
EB+AND	71.92 ± 2.87b	6.64 ± 0.85b	16.62 ± 2.81ab	3.70 ± 0.51b	485.68 ± 17.46b
M + EB + AND	74.45 ± 6.43b	5.23 ± 0.30b	20.74 ± 5.42a	3.70 ± 0.1b	483.44 ± 16.83b

CF, control plants with no organic amendment and conventional inorganic fertilization; M + CF, manure pellet amended soil and conventional fertilization; EB+AND, enriched‐biochar amended soil and organic fertilizer AND; M + EB + AND, manure pellet and enriched‐biochar amended soil treated with organic fertilization AND. Numbers show average value per treatment in the eight randomized subplots (*n* = 8) ± standard deviation. One‐way analysis of variance (ANOVA) following by multiple‐range Tukey's test was used to separate means. Different letters indicate statistically significant differences (*P* < 0.05).

Regarding the percentage of flowering heads or stalks that were refused due to their no‐commercial characteristics (hollow stem, too small size, etc.), CF and M + CF showed a percentage of 30%, following by M + EB + AND treatment, where 22% of the flowering heads where discarded, and EB+AND treatment with 18% of refused flowering heads (Fig. 4B).

The fresh weight of the flowering heads (FWs) showed that CF treatment presented the highest FWs (Table 1) and no significant differences were found in FWs for M + CF, EB+AND and M + EB + AND treatments.

### Total leaf fresh weight and dry weight

There were no significant differences in the canopy fresh weight between treatments (Table 2). The lowest dry weight of the total leaf biomass was for the M + CF treatment. A high correlation was found (*R* = 0.87) between the dry weight of the canopy and the N content in the leaves (data not shown).

**Table 2:** Fresh weight and dry weight of the leaves

Treatment	Leaves FW (g)	Leaves DW (g)
CF	1892 ± 87a	226 ± 17a
M + CF	1650 ± 226a	168 ± 28c
EB+AND	1992 ± 52a	214 ± 2b
M + EB + AND	2047 ± 226a	213 ± 12b

CF, control plants with no organic amendment and conventional inorganic fertilization; M + CF, manure pellet amended soil and conventional fertilization; EB+AND, enriched‐biochar amended soil and organic fertilizer AND; M + EB + AND, manure pellet and enriched‐biochar amended soil treated with organic fertilization AND. Numbers show average value per treatment in the eight randomized subplots (*n* = 8) ± standard deviation. One‐way analysis of variance (ANOVA) following by multiple‐range Tukey's test was used to separate means. Different letters indicate statistically significant differences (*P* < 0.05).

### Glucosinolates

**Figure 5:**
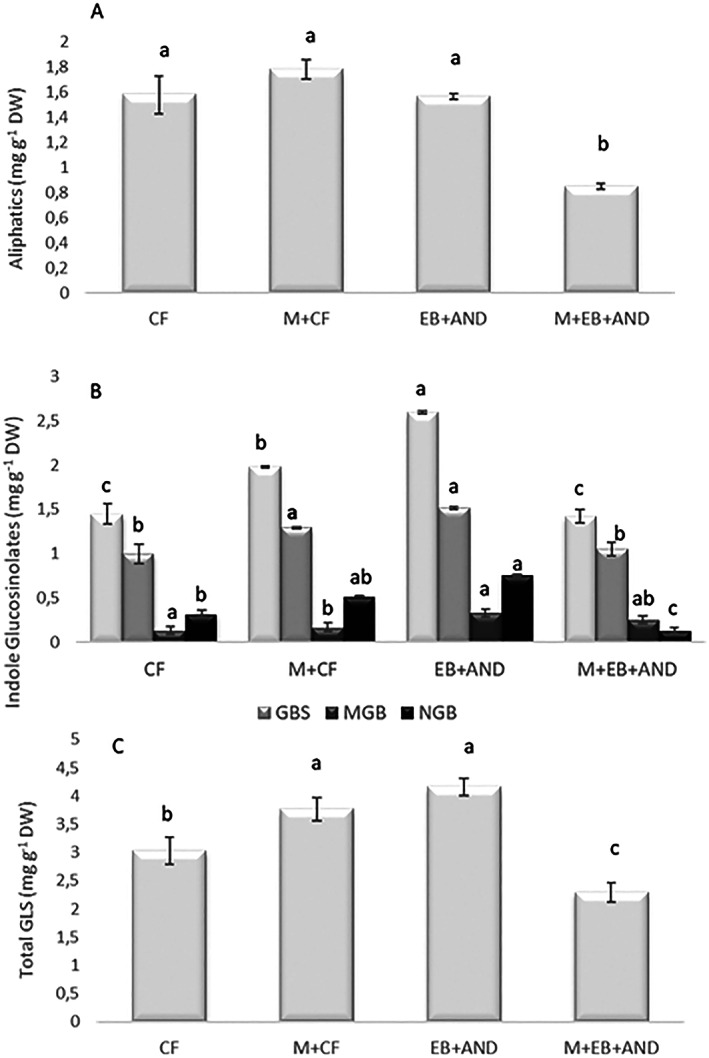
Aliphatic (A), indole (B) and total GSL content (C) in the broccoli flowering head of plants treated with: CF, control plants with no organic amendment and conventional inorganic fertilization; M + CF, manure pellet amended soil and conventional fertilization; EB+AND, enriched‐biochar amended soil and organic fertilizer AND; M + EB + AND, manure pellet and enriched‐biochar amended soil treated with organic fertilization AND. GRA, glucoraphanin; HGB, 4‐hydroxyglucobrassicin; GB, glucobrassicin; MGB, 4‐metoxyglucobrassicin; NGB, neoglucobrassicin. Numbers show average value per treatment (*n* = 8) ± standard deviation. Different letters indicate statistically significant differences (*P* < 0.05).

GSL quantification was determined in broccoli flowering heads (Fig. 5). The only aliphatic GSL detected was glucoraphanin (GRA) (Supporting Information, Fig. S1). No significant differences were found for GRA in all treatments except in M + EB + AND treatment, where the amount of GRA was the lowest (Fig. 5A). The sum of indolic GSL (4‐hydroxyglucobrassicin (HGB); glucobrassicin (GB); 4‐metoxyglucobrassicin (MGB); neoglucobrassicin (NGB)) was calculated (Fig. 5B). HGB could not be quantified in any treatment in these flowering heads. A statistically significant higher content in total indolic GSLs was observed in plants grown on M + CF and EB+AND treatments regarding CF, while M + EB + AND treatment showed no significant differences in the total indolic GSL regarding CF. Similar results were found for GBS that was higher in the M + CF and EB+AND treatments, while M + EB + AND treatment showed no differences regarding CF. MGB content was only higher in the EB+AND treatment in comparison with the rest of the treatments. Finally, NGB content was higher in the EB+AND treatment and lower in the M + EB + AND treatment with regard to CF. In general, the application of amendments applied alone in the forms of manure pellet (M + CF) or biochar (EB+AND) resulted in higher total GSL content compared to CF (Fig. 5C), but when amendments were applied in combination (M + EB + AND) the GSL content was lower. The total GSL concentration content in broccoli flowering heads under M + CF and EB+AND treatments was driven by a sharp increase in GBS and NGB, which were significantly higher than in the rest of the treatments.

### Phenolic compounds

**Figure 6:**
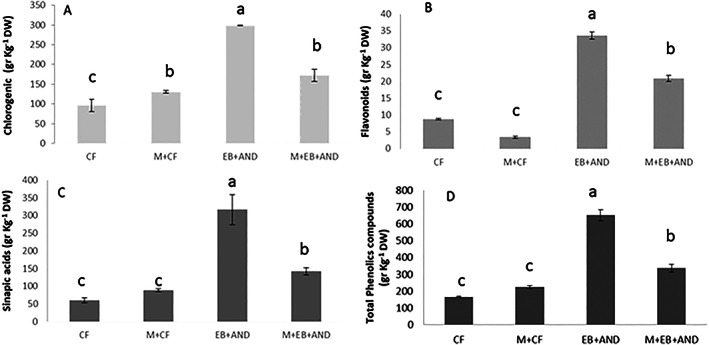
Phenolic content. Chlorogenic (A) and sinapic acid (B) derivatives, flavonoids (C) and total phenolic compounds (D) in broccoli flowering heads of plants treated as follows: CF, control plants with no organic amendment and conventional inorganic fertilization; M + CF, manure pellet amended soil and conventional fertilization; EB+AND, enriched‐biochar amended soil and organic fertilizer AND; M + EB + AND, manure pellet and enriched‐biochar amended soil treated with organic fertilization AND. Numbers show average value per treatment (*n* = 8) ± standard deviation. Different letters indicate statistically significant differences (*P* < 0.05).

Phenolic compounds were determined and classified into three subtypes: chlorogenic acid derivate, flavonoids and sinapic acid derivate (Fig. 6). As evidence by Fig. 6(A), the manure (M + CF) and biochar amendments (EB+AND and M + EB + AND) resulted in increased chlorogenic acids compared to CF, the content being higher in the EB+AND treatment. Regarding flavonoids, only biochar treatments (EB+AND and M + EB + AND) procured higher levels of these phenolic compounds with regard to CF (Fig. 6B). The treatment M + CF resulted in the lowest flavonoid content. Similar results were found for sinapic acids, and EB+AND and M + EB + AND treatments increased their content with regard to CF and M + CF treatments, which showed similar values (Fig. 6C). Finally, the total phenolic content was higher in biochar amendment treatments (EB+AND and M + EB + AND) (Fig. 6D).

### Water (WUE), phosphate (PUE) and nitrogen (NUE) use efficiency

**Figure 7:**
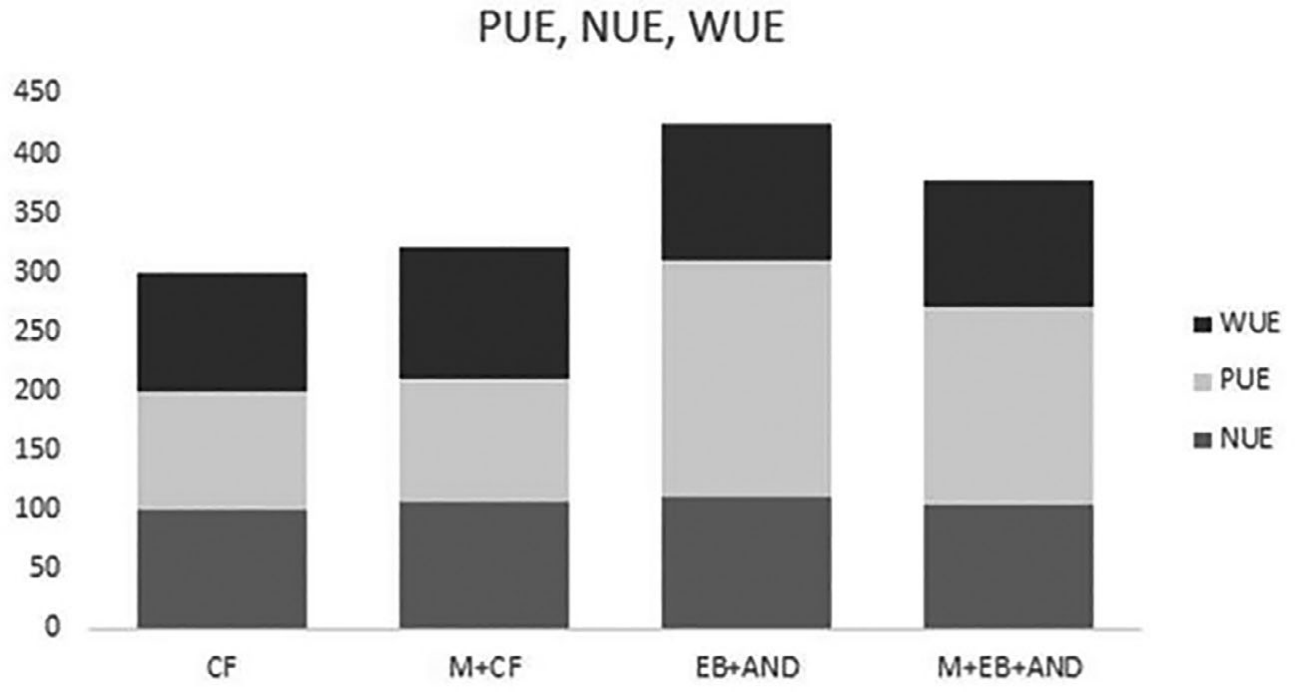
Water (WUE), phosphate (PUE) and nitrogen (NUE) use efficiency expressed as a percentage with regard to conventional fertilization (CF). CF, control plants with no organic amendment and conventional inorganic fertilization; M + CF, manure pellet amended soil and conventional fertilization; EB+AND, enriched‐biochar amended soil and organic fertilizer AND; M + EB + AND, manure pellet and enriched‐biochar amended soil treated with organic fertilization AND.

WUE, PUE and NUE were calculated as yield (kg m^−2^) per amount of water (m^3^) for irrigation, phosphorus (kg m^−2^) and nitrogen (kg m^−2^) respectively, and expressed as percentage with regard to CF, which was considered 100% (Fig. 7). It has been shown that WUE was similar in all treatments, but the maximum values were for EB+AND treatment, followed by M + CF and M + EB + AND compared with CF. A marked difference between treatments was found in PUE, where EB+AND showed the maximum value, followed by M + EB + AND compared with CF and M + CF. The highest NUE value was for EB+AND treatment, whereas no significant differences were found for CF, M + CF and M + EB + AND treatments.

### Flowering head cations and anions

Flowering head cations and anions are shown in Table 3. Mg^2+^ was similar in all treatments except in M + CF treatment, where the content of Mg^2+^ was significantly lower with regard to the rest of the treatments. Regarding Ca^2+^ content, no significant differences between treatments were observed, but there was a trend to accumulate lower Ca^2+^ in the flowering heads of M + CF and EB+AND treatments. The highest K^+^ contents were found in both enriched‐biochar treatments, EB+AND and M + EB + AND, whereas in the M + CF treatment the lowest content was shown compared to CF. NH_4_^+^ levels were reduced in a similar way in all treatments with regard to CF, but this treatment showed the lowest NH_4_^+^ content. Finally, Na^+^ was higher in EB+AND and M + EB + AND treatments with regard to CF and M + CF‐ treated plants.

**Table 3:** Cations and anions (mg kg^−1^ FW) in flowering heads of different treatments

Treatment	Cation				
	Mg^2+^	Ca^2+^	K^+^	NH_4_^+^	Na^+^
CF	219.23 ± 12.64a	464.03 ± 22.12a	3116.28 ± 75.95b	71.65 ± 2,92a	314.28 ± 2.25b
M + CF	179.82 ± 6.55b	420.53 ± 24.73a	2836.01 ± 31.66c	54.55 ± 8.94 b	283.87 ± 4.11b
EB+AND	225.43 ± 8.10a	417.20 ± 37.27a	3494.09 ± 16.14a	49.25 ± 3.76 b	347.34 ± 5.94a
M + EB + AND	200.76 ± 6.44ab	484.24 ± 32.26a	3315.69 ± 59.82a	47.88 ± 11.24 b	357.72 ± 8.82a

Treatment	Anion				
	Cl^−^	NO_2_^−^	NO_3_^−^	PO_4_^3−^	SO_4_^2−^
CF	225.77 ± 3.83a	32.77 ± 0.36b	408.58 ± 7.53b	2143.72 ± 33.08b	1246.14 ± 17.28b
M + CF	155.28 ± 2.93b	40.02 ± 2.94a	362.20 ± 5.70c	2116.81 ± 12.64b	1131.00 ± 10.08b
EB+AND	232.97 ± 15.06a	35.40 ± 0.49b	497.90 ± 33.34a	2485.83 ± 59.88a	1460.25 ± 44.89a
M + EB + AND	258.92 ± 25.01a	34.64 ± 0.30b	521.03 ± 26.21a	2328.08 ± 11.87a	1370.96 ± 24.79a

CF, control plants with no organic amendment and conventional inorganic fertilization; M + CF, manure pellet amended soil and conventional fertilization; EB + AND, enriched‐biochar amended soil and organic fertilizer AND; M+EB + AND, manure pellet and enriched‐biochar amended soil treated with organic fertilization AND. Numbers show average value per treatment (*n* = 8) ± standard deviation. One‐way analysis of variance (ANOVA) followed by multiple‐range Tukey's test was used to separate means. Different letters indicate statistically significant differences (*P* < 0.05).

For anions, the content of Cl^−^ was similar for all treatments, except for M+, where the concentration of this ion was the lowest. By contrast, M + CF treatment presented the highest NO_2_^−^ compared with the rest of the treatments. NO_3_^−^ was higher in both biochar treatments, EB + AND and M+EB + AND, with regard to CF, whereas it was reduced in M + CF treatment. Finally, PO_4_^3−^ and SO_4_^2−^ contents showed similar variations, and both anions were significantly higher in EB + AND and M+EB + AND treatments in comparison to CF and M + CF treatments, which had similar ion content.

### Total carbon, nitrogen and sulfur in the plant

Total percentages of carbon (C), nitrogen (N) and sulphur (S) as well as the C/N and N/S relations were determined in broccoli flowering heads (Table 4). Similar percentage values for C were found in all treatments, whereas N percentage was lower for all treatments with regard to CF, the lowest value being in the M + CF treatment, followed by EB + AND and M+EB + AND treatments, respectively. The C/N relation was, by contrast, higher in M + CF treatment with regard to CF, followed by EB + AND and M+EB + AND, in consonance with N values. Sulfur percentage was similarly higher in EB + AND and M+EB + AND treatments compared to CF, whereas no significant differences were observed in CF and M + CF treatments. The N/S relation in the flowering heads was lower for all treatments with regard to CF, the minimum value being for the M + CF treatment, followed by EB + AND and M+EB + AND, respectively.

**Table 4:** C/N relation, C, N and S percentage and N/S ratio in the flowering heads of different treatments

Treatment	C/N	C (%)	N (%)	S (%)	N/S
CF	3.56 ± 0.17d	40.10 ± 0.10a	10.35 ± 0.04a	0.35 ± 0.04a	29.57 ± 1.45a
M + CF	8.79 ± 0.30a	40.01 ± 0.22a	4.23 ± 0.22d	0.38 ± 0.05ab	11.13 ± 0.65d
EB + AND	6.33 ± 0.21b	40.08 ± 0.20a	6.36 ± 0.24c	0.41 ± 0.05b	15.9 ± 0.85c
M+EB + AND	4.77 ± 0.22c	40.50 ± 0.17a	8.56 ± 0.44b	0.40 ± 0.06b	25.17 ± 1.24b

CF, control plants with no organic amendment and conventional inorganic fertilization; M + CF, manure pellet amended soil and conventional fertilization; EB + AND, enriched‐biochar amended soil and organic fertilizer AND; M+EB + AND, manure pellet and enriched‐biochar amended soil treated with organic fertilization AND. Numbers show average value per treatment (*n* = 8) ± standard deviation. One‐way analysis of variance (ANOVA) following by multiple‐range Tukey's test was used to separate means. Different letters indicate statistically significant differences (*P* < 0.05).

## DISCUSSION

The effect of organic amendments on yield has been determined in different reports. It has been shown that biochar and compost effects on plant growth and yield were remarkable when they were applied in combination with nitrogen fertilization.^28^ In our experiment, the replacement of inorganic nitrogen fertilizer with an enriched‐biochar amendment with organic fertilization had a positive impact on crop yield. However, no synergistic effect of the combined manure pellet and enriched‐biochar on crop yield was observed. By contrast, in different reports it has been observed that composted‐biochar had an important effect on plant growth promotion compared with the use of pure biochar.^29^ In the literature, however, the results are contradictory and many authors found antagonistic interactions between organic amendment–biochar mixtures.^30^ Differences were due mainly to the distinct nature of the organic materials, soil characteristics and field experimental conditions. Thus it has been demonstrated that nitrogen‐enriched materials such as compost^31^ or urine,^32^ which contribute 60 kg ha^−1^ of organic N, may promote synergistic effects on plant growth when soil pH ranges from 5 to 7. In our case, manure pellet had a lower organic N contribution of 40 kg ha^−1^ and the combination with enriched‐biochar was not effective in terms of plant yield under an elevated soil pH of 8.7.

Also, an over‐fertilization or another limiting factor in the manure‐enriched biochar plot cannot be discarded. In fact, higher fresh weight and head diameters were found in CF flowering heads compared to the rest of the treatments, indicating that the increase in yield observed with enriched‐biochar amendment was due to a reduction of the refused flowering heads as a consequence of no‐commercial characteristics.

GSLs and their degradation products play important roles in abiotic and biotic stress tolerance, playing an important role as signalling molecules and affecting plant physiology.^33^

S fertilization and its interaction with N fertilization have been reported to play an important role in GSL concentration in brassica vegetables. Rather than only S or N fertilization,^34^ a negative linear relationship has been observed between total GSL concentration and the N/S ratio in the leaves of two brassica cultivars.^35^ In our broccoli flowering heads a higher correlation (*R* = 0.97) was found between total GSLs and S, rather than total GSLs and the N/S ratio, which were inversely correlated (*R* = −0.74). This indicates that S is a main determinant of the concentration of total GSLs in the flowering heads of broccoli plants grown under our experimental conditions, taking into consideration that in all parcels the soil sulfur content was low. As N/S ratio increased, the GSL content decreased, probably due to the vegetative growth of the plant according to the enhanced N content, which interferes with the secondary metabolism of the plant.^36^ Thus a linear correlation (*R* = 0.77) was found between total leaf dry weight and N content in the leaf tissues (data not shown), but other factors regulating GSL content in addition to the N/S ratio cannot be ruled out.

Enriched‐biochar treatment (EB + AND) increased the total indole GSL with regard to CF and M + CF treatments and therefore total GSLs. This result is in accordance with previous findings where the application of olive tree pruning biochar to broccoli plants increased total GSL content.^22^ It has been reported that biochar alters soil chemical and biological characteristics and the production of indole GSL, which is mainly regulated by environmental conditions rather than by genetic factors, as occurred in the production of aliphatic GSL.^37^ Our results are in consonance with this fact.

However, when enriched‐biochar was applied together with organic manure (M+EB + AND), the GSL content was decreased compared to application of enriched‐biochar only. By contrast, it has been recently reported that *Melastoma malabathricum* L. plants amended with food waste compost had higher secondary metabolites, (phenol, flavonoids, alkaloids, saponin and tannins) in the leaves and roots compared to those amended with palm kernel biochar.^38^ However, the interaction of both amendments was not studied, and the nature of secondary metabolites differs from that studied here. To the best of our knowledge, there is a lack of studies related to the effect of biochar and other organic amendments interaction on vegetable/fruit bioactive compounds.

In any case, the GSL content of the flowering heads is relatively low compared to other broccoli cultivars,^27^ and a genotypic variation has to be taken into account. Rios *et al*. studied the GSL content of seven different broccoli landraces at distinct growing seasons (spring, autumn and winter).^39^ The authors showed that in addition to the genotypic response an increased level of GSLs was observed during spring in all varieties. Changes in temperature and day length were involved in broccoli response in relation to yield and GSLs. These environmental changes could also modulate the yield and GSL response in our broccoli plants.

Also, GSL concentration in the plant depends on S fertilization, and the soil of our plots contained poor sulfate levels. However, the addition of only enriched‐biochar increased GSL content compared to the rest of the treatments promoting the functional value of the flowering heads, since GSLs have been shown to be precursor compounds (isothiocyanates) involved in prevention of different diseases such as cancer and inflammatory diseases,^40^ and its content under organic amendment is of importance.

Different polyphenolic structures influence plant stress responses.^41^ Total phenolic compounds were determined, separating into the classes of phenolic compounds present in broccoli (chlorogenic and sinapic acid derivatives and flavonoids); this showed that sinapic acids were the major group of phenolics in all the treatments. The higher content of all phenolic compounds after enriched‐biochar amendment indicated that this treatment is a valuable tool to increase high‐nutritional and functional quality broccoli in the market, and from a nutraceutical point of view it is important to consider these antioxidant characteristics.

Similar results were found in pak choi (*Brassica rapa* L. cultivar group Pak choi, Green‐Petioled Form) plants, where biochar treatment increased crop bioactive compounds such as GSL and phenolic compounds. Also, in *Arabidopsis thaliana* anthocyanins and flavonols were increased after biochar application.^42^ The addition of biochar to contaminated soil with copper restored (flavonoids) or exceeded (total phenols, phenolic acids and anthocyanins) control values in lettuce plants, which was correlated with higher antioxidant activity. However, in this case the amendment did not influence the composition of the different phenolic classes, indicating that the genotype and experimental conditions may influence differentially in relation to distinct phenolic compounds.

Some authors have suggested a bio‐stimulant effect of biochar,^43^, ^44^ and this could be our case due to the effect of biochar bacteria, but the mechanisms involved in the phenolic compounds regulation after biochar addition are not clear and need to be elucidated.

Different nutritional status was observed among treatments in the broccoli flowering heads. It has been reported that broccoli constitutes an essential dietary source of some macro‐ and micronutrients.^45^ Ca^2+^ content in the different treatments were in the range of concentrations found for this ion in different broccoli cultivars,^45^, ^46^, ^47^ indicating that broccoli flowering heads under the different amendment treatments continue to be a good source of available Ca^2+^ for human nutrition.

The higher Na^+^ content in the flowering heads after amendment application was correlated (*R* = 0.70) with the gradual increase of assimilable Na^+^ in the soil imposed by the treatments. The American Heart Association recommends no more than 2300 mg per day; therefore 100 g of this fresh broccoli flowering heads may contribute within the range of 12.2–15.2% mg to the recommended daily allowance (RDA). The Na^+^ content falls in the range of 30–192 mg 100 g^−1^ FW in other *Brassica oleracea* vegetables.^47^, ^48^, ^49^ In *Brassica oleracea* var. capitata L., an Na^+^ content of 176.00 mg 100 g^−1^ FW was described.^50^ Therefore, our Na^+^ content is lower compared with other brassica vegetables, even if enriched‐biochar with organic fertilization increased the assimilable Na^+^ soil level.

In the enriched‐biochar plots increased soil and plant K^+^ content has been observed in relation to the CF treatment. However, rather than a biochar effect this fact could be associated with the imposed organic fertilizer. The K^+^ in the soils of EB + AND and M+EB + AND plots was 535 and 593 mg kg^−1^ respectively, compared to CF (449 mg kg^−1^) (Supporting Information, Table S6), coinciding with a K^+^ increase in the flowering heads of these two treatments (EB + AND and M+EB + AND). However, a clear correlation between the soil and plant K^+^ concentrations cannot be established for all treatments. Zhang *et al*. also found an increased root system promoted by biochar, which was not observed in the case of only organic manure addition, influencing differentially the nutrient uptake.^51^ If an analogous effect on our broccoli root system occurred after enriched‐biochar addition must be explored. In fact, similarly to plant K^+^ content, PO_3_^4−^, NO_3_^−^ and SO_4_^2−^ contents resulted increased in the broccoli flowering heads after enriched‐biochar (EB + AND and M+EB + AND) treatments in comparison with CF and M + CF treatments. In any case, these results indicated the efficiency of enriched‐biochar addition in nitrogen and phosphate plant uptake and their influence on total crop yield.

From a nutritional point of view, the K^+^ content is in the range of other broccoli cultivars.^45^ Also, the NO_3_^−^content is in the range of those levels founds in broccoli florets (558 mg kg^−1^ FW)^48^ and lower compared to those found in different cruciferous species, such as radish (2030 mg kg^−1^ FW) or lettuce crops (1489.0 mg kg^−1^ FW). The *EFSA Journa*l reports that brassica vegetables contained the lowest nitrate concentration (from 24 mg kg^−1^ nitrates in Brussels sprouts to 987 mg kg^−1^ nitrates in kohlrabi) compared to other vegetables, and it seems that they do not present toxicological effects on human health, independent of their agriculture management.^52^

Worthington compared conventional *versus* organic fertilization in terms of better vegetable or fruit quality, considering a high number of reports.^53^ In general, no significant differences were found in the mineral content of the vegetables/fruits grown under the two fertilization methods (conventional *versus* organic fertilization), with the exception of magnesium (Mg^2+^), and phosphorus (PO_4_^3−^) contents, which were significantly higher in organic fertilization and nitrates, which were lower compared to conventional crops. A different trend was observed in our broccoli flowering heads, where Mg^2+^ was lower after manure pellets (M + CF) amendment, and combined manure pellets and enriched‐biochar treatments (M+EB + AND) showed a content of Mg^2+^ similar to CF flowering heads. The nature of the organic treatment may influence the results, but in our case an increased Mg‐cation exchange capacity and soil‐assimilable Mg^2+^ were also found in the soil after the enriched‐biochar amendments (EB + AND and M+EB + AND), (Supporting Information, Table S6). However, a direct relation between soil Mg^2+^ content and its concentration in the flowering heads cannot be established for all treatments, and other edaphoclimatic factors must be considered in the plant Mg uptake. The genetic influence in Mg^2+^ content was analysed in different hybrid broccoli heads^54^ ranging from a content of 200 to 350 mg kg^−1^, which was in agreement with the levels found in our broccoli flowering heads under all treatments.

## CONCLUSIONS

Enriched‐biochar application to the soil enhanced the crop yield and reduced the number of flowering heads with no‐commercial attributes. Also, enriched‐biochar increased the content of some minerals (NO_3_^−^ and PO_4_^3−^), indicating its efficiency in nitrogen and phosphorus plant uptake and the influence of this nutrition on total crop yield.

Phenolic compounds and indolic GSL were also enhanced after enriched‐biochar application, the increment being higher than that induced by organic manure, and a bio‐stimulant effect of enriched‐biochar cannot be discarded. The GSL increment was related to an increased percentage of sulfur in the flowering heads rather than an adequate N/S ratio. The combination of enriched‐biochar with manure amendment did not result in synergistic effects on crop yield and nutritional parameters. Therefore, enriched‐biochar application may improve minerals and bioactive compounds in broccoli flowering heads under field cultivation without a loss of productivity.

## CONFLICT OF INTEREST

The authors declare no conflict of interest.

## Supporting information


**Table S1.** Fertilization planning for each treatment; CF: control plants with no organic amendment and conventional inorganic fertilization; M + CF: manure pellet amended soil and conventional fertilization; EB+AND: enriched‐biochar amended soil and organic fertilizer AND; M + EB + AND: manure pellet and enriched‐biochar amended soil treated with organic fertilization AND.
**Table S2**. Nutrient composition of manure pellet (M)
**Table S3.** Nutrient composition of organic fertilizer AND.
**Table S4.** Nutrient composition of enriched‐biochar (EB)
**Table S5.** Category flowering heads classification according to the size, stem and grain characteristics.
**Table S6.** Physicochemical properties of the soil for each treatment plot. The samples were collected at two depths (30 and 50 cm). CF: control plants with no organic amendment and conventional inorganic fertilization; M + CF: manure pellet amended soil and conventional fertilization; EB+AND: enriched‐biochar amended soil and organic fertilizer AND; M + EB + AND: manure pellet and enriched‐biochar amended soil treated with organic fertilization AND.
**Figure S1**. HPLC chromatograms for glucosinolate determination. CF: control plants with no organic amendment and conventional inorganic fertilization; M + CF: manure pellet amended soil and conventional fertilization; EB+AND: enriched‐biochar amended soil and organic fertilizer AND; M + EB + AND: manure pellet and enriched‐biochar amended soil treated with organic fertilization AND. GRA: glucoraphanin; HGB: 4‐hydroxyglucobrassicin; GB: glucobrassicin; MGB: 4‐metoxyglucobrassicin; NGB: neoglucobrassicin.
