# Supplementary material for: Enriched‐biochar application increases broccoli nutritional and phytochemical content without detrimental effect on yield
Source: J Sci Food Agric. 2022 Jul 28;102(15):7353–62. doi: 10.1002/jsfa.12102 (PMC9796967; doi:10.1002/jsfa.12102)
Supplement: Supplementary file 1 — Table S1. Fertilization planning for each treatment; CF: control plants with no organic amendment and conventional inorganic fertilization; M + CF: manure pellet amended soil and conventional fertilization; EB+AND: enriched‐biochar amended soil and organic fertilizer AND; M + EB + AND: manure pellet and enriched‐biochar amended soil treated with organic fertilization AND. Table S2. Nutrient composition of manure pellet (M) Table S3. Nutrient composition of organic fertilizer AND. Table S4. Nutrient composition of enriched‐biochar (EB) Table S5. Category flowering heads classification according to the size, stem and grain characteristics. Table S6. Physicochemical properties of the soil for each treatment plot. The samples were collected at two depths (30 and 50 cm). CF: control plants with no organic amendment and conventional inorganic fertilization; M + CF: manure pellet amended soil and conventional fertilization; EB+AND: enriched‐biochar amended soil and organic fertilizer AND; M + EB + AND: manure pellet and enriched‐biochar amended soil treated with organic fertilization AND. Figure S1. HPLC chromatograms for glucosinolate determination. CF: control plants with no organic amendment and conventional inorganic fertilization; M + CF: manure pellet amended soil and conventional fertilization; EB+AND: enriched‐biochar amended soil and organic fertilizer AND; M + EB + AND: manure pellet and enriched‐biochar amended soil treated with organic fertilization AND. GRA: glucoraphanin; HGB: 4‐hydroxyglucobrassicin; GB: glucobrassicin; MGB: 4‐metoxyglucobrassicin; NGB: neoglucobrassicin. [file JSFA-102-7353-s001.docx]

|  | Ademenment (Kg/Ha) | | Fertilization (Kg/Ha) | | | | |
| --- | --- | --- | --- | --- | --- | --- | --- |
| **Treatments** | Manure pellet | Enriched-Biochar | Calcium Nitrate | Potassium Nitrate | Monoammonium Phosphate | Magnesium nitrate | AND |
| **CF** | - | - | 80 | 210 | 300 | 335 | - |
| **M+CF** | 15.000 | - | 80 | 210 | 300 | 335 | - |
| **EB+AND** | - | 10.000 | - | - | - | - | 1980 |
| **M+EB+AND** | 15.000 | 10.000 | - | - | - | - | 1980 |

**Supplemental Table 1.** Fertilization planning for each treatment; CF: Control plants with no organic amendment and conventional inorganic fertilization; M+CF: Manure pellet amended soil and conventional fertilization; EB+AND: Enriched-biochar amended soil and organic fertilizer AND; M+EB+AND: Manure pellet and enriched-biochar amended soil treated with organic fertilization AND.

**Supplemental Table 2**. Nutrient composition of Manure pellet (M)

| **Manure pellet composition** | pH | N (%) | P_2_O_5_ (%) | K_2_O (%) | CaO (%) | MgO (%) | Organic matter | Carbon in the dry weight of Charcoal |
| --- | --- | --- | --- | --- | --- | --- | --- | --- |
| **M** | 6,5 | 1,1 | 1,7 | 1,9 | 0 | 0 | 33,0% | 19,10% |

**Supplemental Table 3.** Nutrient composition of organic fertilizer AND.

| **Organic fertilizer** | pH | N (%) | P_2_O_5_ (%) | K_2_O (%) | CaO (%) | MgO (%) | Organic matter |
| --- | --- | --- | --- | --- | --- | --- | --- |
| **AND** | 5-6 | 4,5 | 0,6 | 3,9 | 0,5 | 0,08 | 52,2% |

**Supplemental Table 4.** Nutrient composition of enriched-biochar (EB)

| **Enriched Biochar** | pH | N (%) | P_2_O_5_ (%) | K_2_O (%) | CaO (%) | MgO (%) | Organic matter | Carbon in the dry weight of Charcoal | microorganism |
| --- | --- | --- | --- | --- | --- | --- | --- | --- | --- |
| **EB** | 6,6 | 2 | 0,3 | 2 | 0,6 | 0,05 | 73,0% | >80% | lactic acid bacteria, photosynthesis bacteria, yeasts |

**Suplemental Table 5.** Category flowering heads classification according to the size, stem and grain characteristics.

| **Broccoli inflorescence category** | **Inflorescence weight and quality parameters** |
| --- | --- |
| First | 225-600 gr normal and tight stem, strong and green grain |
| Second | 601-800 gr. normal and tight stem, strong and green grain |
| Industry | + 800 gr. normal and tight stem, strong and green grain |
| Fourth | Leaves, trunks, spiked, withered, rotten, with worm, yellow grain, not compact. |

**Suplemental Table 6.** Physico-chemical properties of the soil for each treatment plot. The samples were collected at two depths (30 and 50 cm). CF: Control plants with no organic amendment and conventional inorganic fertilization; M+CF: Manure pellet amended soil and conventional fertilization; EB+AND: Enriched-biochar amended soil and organic fertilizer AND; M+EB+AND: Manure pellet and enriched-biochar amended soil treated with organic fertilization AND.

|  |  |  |  |  |  |
| --- | --- | --- | --- | --- | --- |
| **Soil Analysis** | **Initial** | **CF** | **M+CF** | **EB+AND** | **M+EB+AND** |
| **pH** | 8,4 | 8,7 | 8,8 | 8,9 | 8,8 |
| **C.E (dS/m)** | 0,535 | 0,342 | 0,347 | 0,4 | 0,491 |
| **Cloruros extrac (meq/L)** | 0,89 | 0,29 | 0,433 | 0,924 | 1,34 |
| **SO_4_^2-^(meq/L)** | 1,49 | 0,727 | 1,03 | 0,52 | 0,895 |
| **Na^+^ (mg/kg)** | 103 | 58,7 | 73,4 | 113 | 152 |
| **NO_3_^-^ (mg/kg N)** | 23,9 | 10,3 | 10,2 | 9,97 | 13,2 |
| **PO_4_^3-^ (mg/kg)** | 219 | 176 | 157 | 167 | 156 |
| **K^+^ (mg/kg)** | 639 | 449 | 481 | 535 | 593 |
| **Ca^2+^ (mg/kg)** | 1490 | 1900 | 1830 | 1780 | 1810 |
| **Mg^2+^ (mg/kg)** | 258 | 264 | 267 | 277 | 273 |
| **CIC (meq/100 g)** | | | | | |
| **Ca^2+^** | 7,16 | 9,06 | 8,76 | 8,55 | 8,64 |
| **Mg^2+^** | 2 | 2,06 | 2,07 | 2,16 | 2,13 |
| **K^+^** | 1,36 | 1,03 | 1,09 | 1,2 | 1,33 |
| **Na^+^** | 0,182 | 0,109 | 0,131 | 0,188 | 0,311 |
| **CIC** | 10,7 | 12,3 | 12,1 | 12,1 | 12,4 |

**Suplemental Figure1**. HPLC-chormatograms for glucosinolates determination. CF: Control plants with no organic amendment and conventional inorganic fertilization; M+CF: Manure pellet amended soil and conventional fertilization; EB+AND: Enriched-biochar amended soil and organic fertilizer AND; M+EB+AND: Manure pellet and enriched-biochar amended soil treated with organic fertilization AND.

GRA: glucoraphanin; HGB: 4-hydroxyglucobrassicin; GB: glucobrassicin; MGB: 4-metoxyglucobrassicin; NGB: neoglucobrassicin.

**
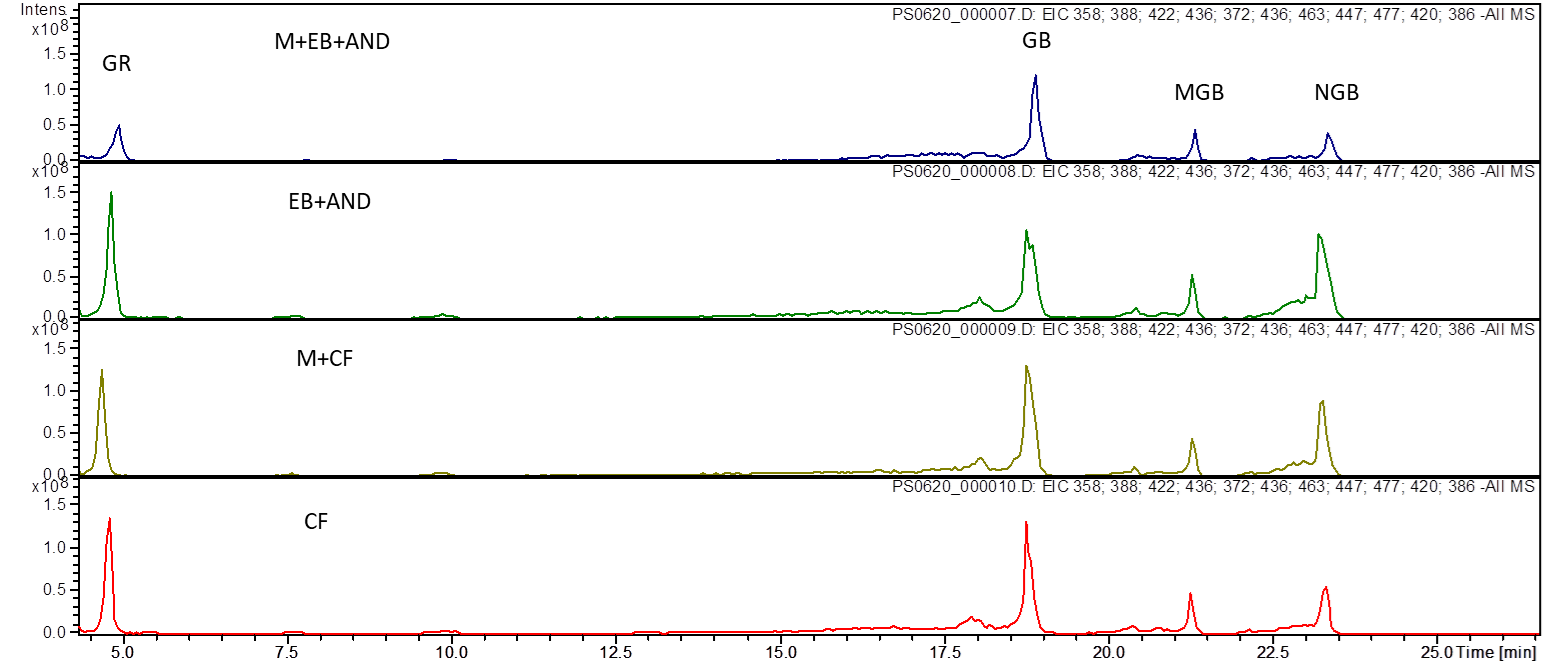
**
